# High-Temperature Properties of Magnesium Ammonium Phosphate Cement Modified with Gold Tailings

**DOI:** 10.3390/ma19122684

**Published:** 2026-06-22

**Authors:** Zhenhai Zeng, Peng Yu, Zhuoyi Chen, Jiale Zhou, Haohui Xin, Lie Yu, Anqing Lin

**Affiliations:** 1 School of Environment and Civil Engineering, Dongguan University of Technology, Dongguan 523808, China; 2 School of Civil and Environmental Engineering, Changsha University of Science and Technology, Changsha 410114, China; 3 School of Civil Engineering, Southeast University, Nanjing 211189, China

**Keywords:** magnesium ammonium phosphate cement, gold tailings, high-temperature performance, strength retention, phase evolution

## Abstract

Magnesium ammonium phosphate cement (MAPC) exhibits rapid setting, high early strength, and potential resistance to elevated temperatures, making it a promising material for rapid repair and fire-resistant applications. Gold tailings (GT), which contain thermally stable Si- and Al-rich components, show potential for improving the high-temperature performance of MAPC. However, the mechanisms by which GT affects the residual performance and phase evolution of MAPC after exposure to elevated temperatures remain insufficiently understood. In this study, GT was used to replace the total binder in MAPC mortar at mass replacement levels of 0%, 10%, 20%, and 30%, while the MgO/NH_4_H_2_PO_4_ mass ratio in the remaining binder was kept constant. The effects of GT content on the workability of MAPC mortar, as well as its visual appearance, mechanical properties, mass loss rate, phase evolution, and microstructure after exposure to elevated temperatures, were investigated. The results showed that GT incorporation shortened the setting time and reduced the fluidity and room-temperature strength. After exposure to elevated temperatures, the GT-containing specimens exhibited higher strength retention and lower mass loss rates. After exposure to 1000 °C, the compressive strength of the specimen containing 30% GT reached 15.37 MPa, which was approximately 44.0% higher than that of the specimen without GT. Its flexural strength retention and mass loss rate were 47.42% and 9.84%, respectively. XRD and SEM results indicated that the formation of high-temperature residual phases, including Mg_3_(PO_4_)_2_, Mg_2_SiO_4_, and aluminosilicates, may contribute to the improvement of the residual matrix structure after exposure to elevated temperatures. Overall, GT incorporation improved the residual mechanical properties of MAPC after exposure to elevated temperatures, and the specimen containing 30% GT showed comparatively superior performance within the experimental scope of this study. These findings provide a reference for the resource utilization of GT in MAPC-based heat-resistant repair materials.

## 1. Introduction

Magnesium phosphate cement (MPC) is an inorganic rapid-hardening cementitious material prepared by mixing dead-burned magnesia (MgO), a soluble phosphate, a retarder, and supplementary materials in specified proportions. Upon the addition of water, MPC can harden rapidly in both air and water [1,2]. Owing to its high early strength, low drying shrinkage, and favorable resistance to elevated temperatures, MPC has potential applications in refractory linings, rapid repair, and post-fire restoration engineering [3,4,5]. Depending on the type of phosphate used, MPC can be classified into magnesium potassium phosphate cement (MKPC) and magnesium ammonium phosphate cement (MAPC). In these systems, MgO reacts with potassium dihydrogen phosphate (KH_2_PO_4_) or ammonium dihydrogen phosphate (NH_4_H_2_PO_4_) through an acid–base neutralization hydration reaction, forming K-struvite (MgKPO_4_·6H_2_O) and struvite (MgNH_4_PO_4_·6H_2_O), respectively [6]. Because potassium salts are relatively expensive [7,8], whereas MAPC offers cost advantages and potential for high-temperature applications, and because studies on the high-temperature modification and evolution of residual properties of MAPC remain limited, MAPC was selected as the focus of this study. The main hydration reaction of MAPC is expressed as follows [9]:

$$\mathrm{MgO}(s)\;+\;{\mathrm{NH}}_{4}H_{2}{\mathrm{PO}}_{4}(s)\;+\;5H_{2}O(l)\;\to \;{\mathrm{MgNH}}_{4}{\mathrm{PO}}_{4}\cdot 6\;H_{2}O(s)$$

Fire can cause severe damage to structures; therefore, MPC, as a rapid repair material, may be exposed to elevated temperatures during service. Previous studies have shown that MPC is a promising refractory cementitious material because of its favorable volume stability and thermal stability [10,11]. When the temperature increases to approximately 1300 °C, MPC undergoes pronounced sintering, leading to substantial strength recovery; however, this process is also accompanied by a linear shrinkage exceeding 10% [12]. In contrast, below 1000 °C, its mechanical properties still deteriorate markedly. Previous studies have reported that the compressive strengths of MPC at 500 °C and 1000 °C are approximately 42% and 36% of its room-temperature strength, respectively [13]. Therefore, clarifying the phase evolution of MPC at elevated temperatures and improving its residual properties and structural stability after exposure to elevated temperatures are of great significance.

Struvite, the main hydration product of MAPC, undergoes dehydration and decomposition during exposure to elevated temperatures, accompanied by the release of volatile components such as NH_3_ and water of crystallization. Subsequently, the decomposition products can be further transformed into magnesium phosphate phases, such as Mg_2_P_2_O_7_ and Mg_3_(PO_4_)_2_ [1,14]. This process destroys the original crystalline binding structure, resulting in an increased mass loss rate and microstructural deterioration, while also involving the reconstruction of high-temperature residual phases. These changes are important factors affecting the residual performance of MAPC after exposure to elevated temperatures. Therefore, it is necessary to further analyze the high-temperature phase evolution and microstructural changes of MAPC after gold tailings (GT) incorporation.

To improve the residual properties of MPC after exposure to elevated temperatures, previous studies have attempted to modify MPC by introducing various mineral or functional components. Metakaolin has been reported to improve the high-temperature mechanical properties of MPC. When 40% of MgO was replaced by metakaolin, the specimen still exhibited strength recovery after exposure to 600 °C [15]. The incorporation of calcium aluminate cement (CAC) can enhance the compressive strength, water resistance, and residual properties of MPC after exposure to elevated temperatures. This improvement is mainly attributed to the encapsulation of K-struvite by CAC hydration products and the formation of MgAl_2_O_4_ at elevated temperatures, which promote matrix densification and enhance thermal resistance [6]. In addition, the incorporation of glass fibers or glass fiber powder can improve the bonding performance of MPC coatings and provide favorable flame retardancy and crack resistance [16]. Yang et al. [10] further reported that the combined incorporation of fly ash and silica fume increased the compressive strength retention of MKPC at 400 °C to 63.3–66.8%, whereas that of the unmodified specimen was only 19.0%. Therefore, mineral materials rich in silicoaluminous components may serve as effective modifiers for the high-temperature modification of MPC. These materials can, to some extent, regulate the high-temperature phase assemblage and microstructure of MPC and mitigate the development of thermal damage, thereby improving its residual properties after exposure to elevated temperatures.

As a typical mining solid waste, tailings are characterized by wide availability, large stockpiles, and an urgent need for resource utilization [17,18]. Owing to their relatively stable mineral composition and fine particle size, tailings can act as fillers, optimize particle grading, and exhibit potential reactivity in cement-based materials [19,20]. GT generally contains abundant Si- and Al-rich components and exhibits a certain degree of stability at elevated temperatures, which may provide skeleton support for the residual structure or participate in partial solid-state reactions [21,22]. Previous studies have shown that GT can be used as an additive or supplementary cementitious material in ordinary Portland cement systems, with potential benefits in reducing binder consumption and improving environmental performance [17,23,24]. Zhang et al. [25] reported that bauxite tailings and their calcined products affected the setting time, fluidity, drying shrinkage, compressive strength, and water resistance of MPC-based materials, indicating that tailing-derived mineral components have potential for performance regulation and resource utilization in phosphate cementitious systems. In addition, Zhu et al. [26] reported that the partial replacement of MgO with GT in MKPC improved mixture workability and promoted the formation of a denser microstructure, thereby enhancing later-age strength. However, MAPC and MKPC differ in their hydration products and high-temperature evolution behavior, and findings obtained from GT-modified MKPC cannot be directly extended to MAPC systems. Therefore, the effects of GT on the residual properties and underlying microstructural mechanisms of MAPC after exposure to elevated temperatures require further investigation.

Because the mechanism by which GT affects the residual properties of MAPC after exposure to elevated temperatures remains unclear, and because its silicoaluminous components may benefit the residual structure after high-temperature treatment, this study investigated the effects of different GT contents on the setting time and fluidity of MAPC mortar. The visual appearance, mechanical strength, and mass loss rate of MAPC mortar after exposure to 300 °C, 600 °C, 900 °C, and 1000 °C were also evaluated. XRD and SEM analyses were further conducted to characterize the phase composition, relative contents of the main phases, and microstructural changes, with the aim of revealing the possible mechanisms by which GT modifies the residual performance of MAPC after exposure to elevated temperatures.

## 2. Materials and Methods

### 2.1. Raw Materials

The main raw materials used in this study included ammonium dihydrogen phosphate, MgO, and borax (Na_2_B_4_O_7_·10H_2_O), all of which were white powders with particle sizes ranging from 75 to 80 μm. The purity of ammonium dihydrogen phosphate was 98%. Borax, with a purity greater than 95%, was used as a retarder. The MgO used in this study was dead-burned magnesia calcined at 1600 °C and had an MgO content of 96.68%. Quartz sand with a particle size of 0.42–2.0 mm was used as the fine aggregate.

**Figure 1:**
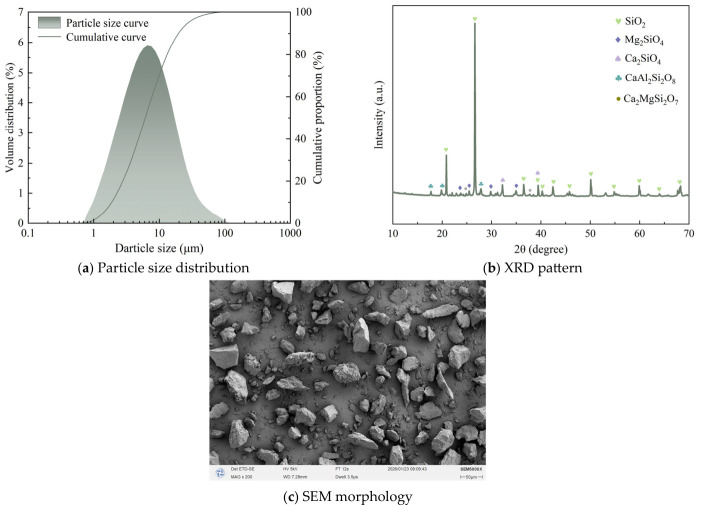
Basic characterization of GT: (**a**) particle size distribution; (**b**) XRD pattern; (**c**) SEM morphology.

The main chemical compositions of GT and MgO are listed in Table 1. The total content of SiO_2_ and Al_2_O_3_ in GT was 85.71%, indicating that GT can be classified as a silicoaluminous mineral additive. The XRD pattern, particle size distribution, and SEM morphology of GT are shown in Figure 1. The D50 of GT was 7.015 μm, and its D10 and D90 were 2.158 μm and 22.487 μm, respectively, suggesting a potential micro-filling effect. SEM observations showed that the GT particles were mostly irregular, blocky, or flaky in shape, with relatively rough surfaces.

### 2.2. Mix Proportions and Specimen Preparation

For the MAPC mortar, the MgO/NH_4_H_2_PO_4_ mass ratio (M/P) was 4, the sand-to-binder ratio (S/C) was 1, and the water-to-binder ratio (W/C) was 0.18, where C denotes the total mass of MgO, NH_4_H_2_PO_4_, and GT. To ensure sufficient working time for specimen preparation, the borax/MgO mass ratio (B/M) was set to 5% [23,27,28]. GT was used to partially replace the total MAPC binder on an equal-mass basis; that is, GT simultaneously replaced MgO and NH_4_H_2_PO_4_, while the MgO/NH_4_H_2_PO_4_ mass ratio in the remaining binder was kept constant. This mixture design was adopted to avoid additional effects of changes in the M/P ratio on the hydration reaction and residual properties after exposure to elevated temperatures. The specimens containing 0%, 10%, 20%, and 30% GT were denoted as G0, G1, G2, and G3, respectively. The detailed mix proportions are presented in Table 2.

During specimen preparation, NH_4_H_2_PO_4_, MgO, GT, borax, and quartz sand were first dry-mixed for 60 s. The specified amount of tap water was then added, followed by further mixing for 120 s. The fresh mortar was cast into molds, demolded after 1 h, and cured at 25 ± 1 °C for 28 d. After curing, the specimens were subjected to room-temperature performance tests and high-temperature treatment.

### 2.3. High-Temperature Exposure Regime

**Figure 2:**
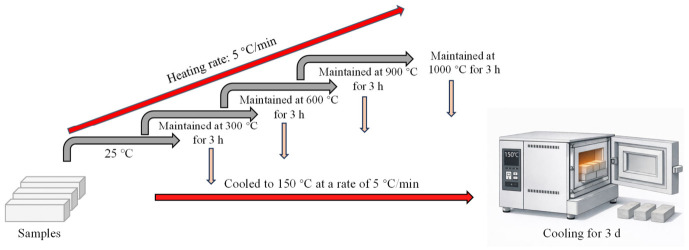
Schematic diagram of the high-temperature furnace heating procedure.

One room-temperature control (25 °C) and four elevated-temperature levels (300 °C, 600 °C, 900 °C, and 1000 °C) were selected to evaluate the changes in the residual properties of MAPC after exposure to elevated temperatures. High-temperature treatment was performed using an NWTX-12A box-type electric furnace (NAWEITE, Luoyang, China). The heating rate was set to 5 °C/min, and the specimens were held at the target temperature for 3 h after the target temperature was reached. Subsequently, the specimens were cooled to 150 °C at a rate of 5 °C/min. The furnace door was then opened to allow natural cooling to room temperature, thereby maintaining a relatively consistent cooling regime. After removal from the furnace, the specimens were stored in a dry laboratory environment for 3 d before strength testing. The heating procedure is illustrated in Figure 2.

### 2.4. Test Methods

#### 2.4.1. Setting Time

The setting time of MAPC was measured using a Vicat apparatus (Wuxi Zhongke Building Materials Co., Ltd., Wuxi, China) in accordance with GB/T 1346-2011 [29] and JC/T 2537-2019 [30]. Because MAPC sets and hardens rapidly, the interval between the initial and final setting times is extremely short. Therefore, the initial setting time was used to characterize the setting time in this study.

#### 2.4.2. Fluidity

The fluidity of MAPC was tested in accordance with JC/T 2537-2019 [30]. Before testing, the flow table and mold were cleaned. The freshly prepared MAPC mortar was rapidly placed into the mold. After the conical mold was lifted, the mortar was allowed to spread freely on the flow table for 30 s. Subsequently, the spread diameters in two mutually perpendicular directions were measured, and their average value was taken as the fluidity.

#### 2.4.3. Mass Loss Rate

The mass of each specimen before heating, $m_{0}$, and the mass after high-temperature treatment and drying, $m_{T}$, were measured using a high-precision electronic balance. The mass loss rate, w, was then calculated according to Equation (1).

(1) $$w=\frac{m_{0}-m_{T}}{m_{0}}\times 100\%$$

#### 2.4.4. Strength Testing

**Figure 3:**
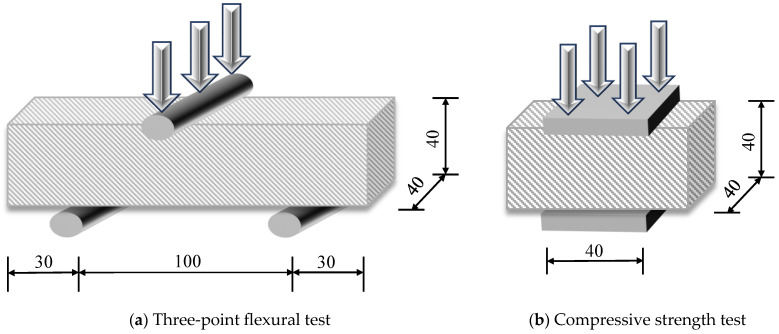
Schematic diagram of strength testing for MAPC specimens: (**a**) three-point flexural strength test; (**b**) compressive strength test. All dimensions are in mm.

The compressive and flexural strengths of the MAPC mortar were tested in accordance with GB/T 17671-1999 [31] using a 100 kN universal testing machine (Jinan Shijin Testing Machine Co., Ltd., Jinan, China). The flexural test specimens had dimensions of 40 mm × 40 mm × 160 mm, with a span of 100 mm, and were loaded at a rate of 0.2 mm/min. Three specimens were tested for each group, and the average value was reported. After the flexural test, the fractured specimens were used for compressive strength testing at a loading rate of 2.4 kN/s. Six specimens were tested for each group, and the average value was reported. A schematic diagram of the strength testing procedure is shown in Figure 3. The mechanical properties and mass loss rate results are expressed as the mean ± standard deviation, and the error bars in the corresponding figures represent the standard deviation.

#### 2.4.5. XRD and SEM Tests

XRD analysis was performed using a Bruker D8 ADVANCE powder X-ray diffractometer (Bruker, Karlsruhe, Germany). The MAPC samples were crushed, ground, and passed through a 75 μm sieve before phase analysis. The 2θ range was 10–70°, with a step size of 0.02° and a scanning rate of 5°/min. Jade 9.0 software was used for phase identification of the representative XRD patterns. Whole-pattern fitting (WPF) and Rietveld refinement were further performed to analyze changes in the relative contents of the main crystalline phases.

SEM analysis was conducted using a ZEISS GeminiSEM 360 scanning electron microscope (ZEISS, Jena, Germany). Representative fragments obtained after strength testing were selected and prepared into small pieces of approximately 1 cm^2^. The samples were sputter-coated with gold before observation, with a sputtering time of 45 s and a sputtering current of 10 mA. This study focused on the typical fracture surface morphologies of G0 and G3 after treatment at different temperatures. Elemental composition analysis was performed on selected regions using energy-dispersive spectroscopy (EDS).

## 3. Results and Discussion

### 3.1. Setting Time and Fluidity

**Figure 4:**
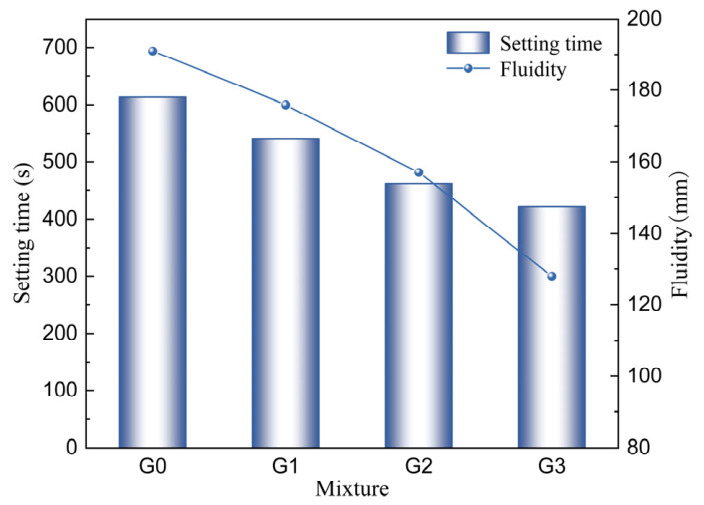
Setting time and fluidity of MAPC specimens.

As shown in Figure 4, the setting time of plain MAPC was approximately 10 min. With GT incorporation, the setting time was markedly shortened. When the GT content increased from 10% to 30%, the reduction in setting time ranged from 11.9% to 31.3%, indicating that GT exerted an accelerating effect on MAPC setting and that this effect became more pronounced with increasing GT content. Previous studies have shown that the setting process of MAPC is closely related to the Mg^2+^ concentration in the system. A higher dissolution rate of MgO leads to a higher Mg^2+^ concentration in the pore solution, thereby accelerating the formation and crystallization of struvite and shortening the setting time of MAPC [32,33]. In addition, alkali metal ions such as K^+^ and Na^+^ may increase the alkalinity of the pore solution and weaken the Mg(OH)_2_ passivation layer on the MgO surface, thereby promoting Mg^2+^ release [34,35,36]. Zhu et al. [26] reported that CaO in GT may consume part of the borax and weaken its retarding effect, which may also contribute to the shortened setting time after GT incorporation.

In terms of fluidity, GT incorporation markedly reduced the fluidity of MAPC mortar. For example, the fluidity of G3 was 128 mm, which was 32.9% lower than that of G0. Based on the particle size distribution and SEM results, GT was mainly composed of micron-sized fine particles, most of which were irregular, blocky, or flaky in shape and had rough surfaces. These characteristics may increase interparticle friction and the water demand of the mixture. In addition, after GT replaced part of the reactive cementitious components, the proportion of filler-type solid particles in the system increased, which also reduced the fluidity of the fresh mixture [37,38].

### 3.2. Visual Appearance

**Figure 5:**
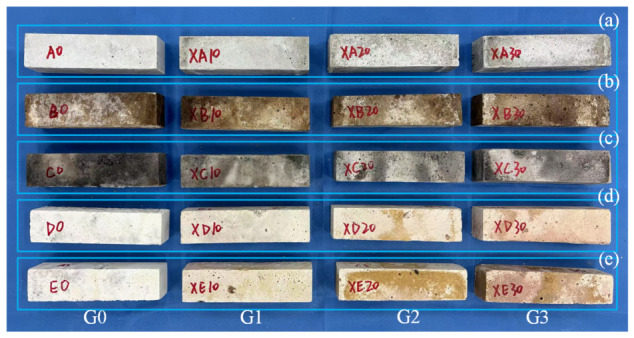
Visual appearance changes of MAPC specimens after treatment at different temperatures: (**a**) 25 °C, (**b**) 300 °C, (**c**) 600 °C, (**d**) 900 °C, and (**e**) 1000 °C.

Figure 5 shows the changes in the visual appearance of MAPC specimens after treatment at different temperatures. At room temperature, all specimens were generally white to light gray. After exposure to 300 °C, the specimen color changed to light brown and became slightly darker with increasing GT content. At this stage, the surfaces of G1 and G2 remained relatively intact, whereas a small number of pores and fine cracks were observed on the surfaces of G0 and G3. These surface defects may be related to the thermal dehydration of struvite and the release of volatile components such as NH_3_ [13,39].

When the temperature increased to 600 °C, the appearance deterioration of the specimens became more evident. Visible cracks and black spots appeared on the surface of G0, and surface cracks also developed in G2 and G3, indicating that the thermal damage of MAPC was aggravated at this stage [40]. After exposure to 900 °C, G0 became whitish, whereas the GT-containing specimens were mostly light yellow, which may be associated with Fe_2_O_3_ and other iron-bearing components in GT. Visible cracks were observed in all groups. When the temperature further increased to 1000 °C, the specimen color became darker, but the surface crack morphology changed only slightly compared with that at 900 °C, indicating that the appearance deterioration tended to level off within this temperature range [5,15,41].

Overall, the color changes of the specimens were mainly governed by temperature, whereas the development of visible cracks and pores was associated with the dehydration and decomposition of struvite, the release of volatile components, and thermal stress. At the same temperature, the GT-containing specimens appeared to exhibit less severe surface deterioration, which may be related to the reduced relative content of decomposable struvite caused by GT incorporation, as well as the potential micro-filling effect of GT and the thermal stability of its silicoaluminous components [42].

### 3.3. Mechanical Properties

#### 3.3.1. Compressive Strength

**Figure 6:**
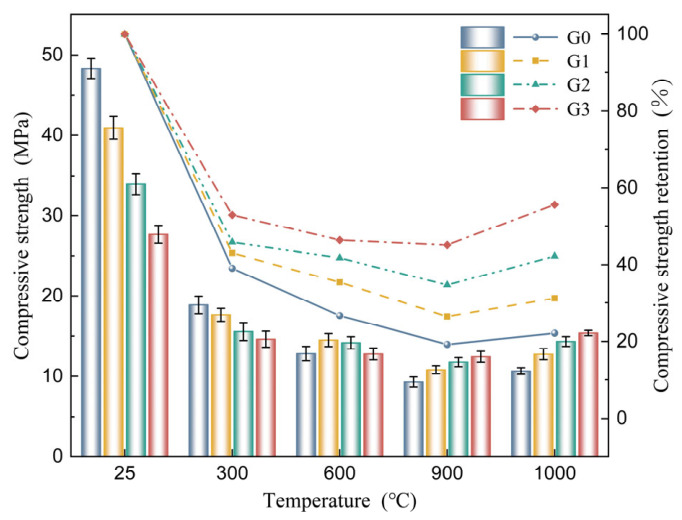
Compressive strength and compressive strength retention of MAPC specimens.

Figure 6 shows the changes in the compressive strength and compressive strength retention of MAPC after treatment at different temperatures. The compressive strength retention was defined as the ratio of the residual compressive strength after treatment at the target temperature to the room-temperature compressive strength of the corresponding group. At room temperature, G0 without GT exhibited the highest compressive strength, reaching 48.28 MPa. As the GT content increased from 10% to 30%, the compressive strength decreased successively to 40.95 MPa, 33.96 MPa, and 27.65 MPa. This was mainly because the equal-mass replacement of part of the total MAPC binder by GT reduced the amounts of reactive MgO and NH_4_H_2_PO_4_ available for hydration, resulting in less formation of the struvite binding phase and thereby weakening the room-temperature compressive strength [39,42]. Nevertheless, the room-temperature compressive strengths of all groups remained higher than 25 MPa, satisfying the basic strength requirement for repair materials [43].

After exposure to elevated temperatures, the strength evolution of MAPC differed markedly from that at room temperature. At 300 °C, the compressive strength of G0 decreased from 48.28 MPa to 18.87 MPa, corresponding to a compressive strength retention of 39.08%. Combined with the mass loss rate results, the strength reduction at this stage was mainly related to the dehydration and decomposition of struvite and the release of volatile components, such as NH_3_ and water of crystallization. These processes damaged the original crystalline binding structure and promoted the development of pores and microcracks [44,45]. In contrast, the compressive strength retention of G3 reached 52.88% at 300 °C. This may be associated with the reduced relative content of decomposable struvite, while the silicoaluminous components in GT may also have had a beneficial effect on the residual structure during the initial stage of exposure to elevated temperatures [46].

When the temperature increased to 600–900 °C, the compressive strength of all groups continued to decrease; however, the compressive strength retention of the GT-containing specimens remained higher than that of G0. At 900 °C, the compressive strength retentions of G0, G1, G2, and G3 were 19.24%, 26.42%, 34.72%, and 45.06%, respectively, indicating that GT incorporation contributed to improving the compressive strength retention of MAPC after exposure to elevated temperatures. At 1000 °C, the compressive strength of all groups recovered to some extent. Previous studies have shown that MPC may undergo residual phase reconstruction, recrystallization, and local sintering after high-temperature treatment, thereby enhancing interparticle bonding and improving the residual matrix structure [40,41]. Notably, G3 exhibited the highest residual compressive strength at 1000 °C, reaching 15.37 MPa, which was approximately 44.0% higher than that of G0. This indicates that, within the experimental scope of this study, the specimen containing 30% GT showed superior residual compressive performance after exposure to elevated temperatures.

#### 3.3.2. Flexural Strength

**Figure 7:**
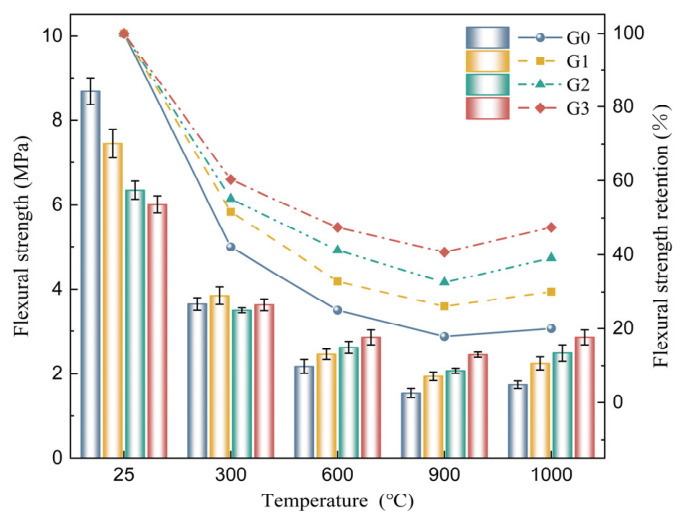
Flexural strength and flexural strength retention of MAPC specimens.

The flexural strength and flexural strength retention of MAPC after treatment at different temperatures are shown in Figure 7. The flexural strength retention was calculated in the same manner as the compressive strength retention. At room temperature, the flexural strength of all specimens decreased with increasing GT content, which was consistent with the trend observed for compressive strength. This indicates that GT incorporation weakened the bonding capacity of the MAPC matrix [5,11]. Flexural strength is more sensitive to microcracks, pores, and interfacial defects, and a relatively weak interfacial zone may have formed between the GT particles and the MAPC matrix. In addition, GT incorporation reduced the fluidity of the mortar, which may have affected the compactness of the molded specimens. Therefore, the room-temperature flexural strength decreased with increasing GT content.

When the temperature increased to 300 °C, the flexural strength of all groups decreased markedly [33,37], indicating that the dehydration and decomposition of struvite during the initial stage of exposure to elevated temperatures weakened the flexural load-bearing capacity of the specimens. Compared with G0, the GT-containing specimens exhibited higher flexural strength retention, suggesting that GT helped mitigate the loss of flexural performance during the initial stage of exposure to elevated temperatures. As the temperature further increased to 600–900 °C, the flexural strength continued to decrease; however, the flexural strength retention of the GT-containing specimens was generally higher than that of G0. At 1000 °C, the flexural strength recovered to some extent. In particular, the flexural strength retention of G3 reached 47.42%, indicating that GT incorporation also had a beneficial effect on retaining the flexural strength of MAPC after exposure to elevated temperatures.

**Figure 8:**
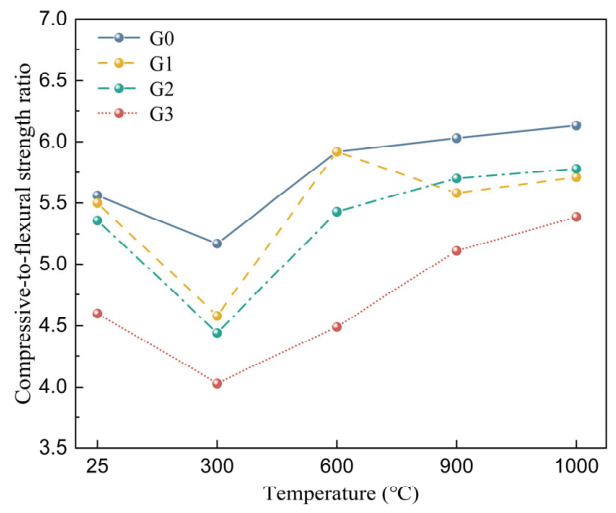
Compressive-to-flexural strength ratio of MAPC specimens.

To further analyze the relationship between compressive and flexural properties, the compressive-to-flexural strength ratio was calculated, as shown in Figure 8. The compressive strength of all specimens was consistently higher than the flexural strength, and both strengths first decreased and then partially recovered with increasing temperature, indicating that they followed similar evolution trends under elevated-temperature exposure. At 300 °C, the strength ratio of each group was slightly lower than that at room temperature, reflecting a more pronounced reduction in compressive strength during the initial stage of exposure to elevated temperatures. When the temperature increased to 600–1000 °C, the ratio generally increased and tended to stabilize, indicating that flexural strength was more sensitive to thermal damage in the medium- and high-temperature ranges. The effects of microcracks and interfacial defects on the flexural load-bearing capacity became more prominent. Overall, the GT-containing specimens maintained relatively high flexural strength retention after exposure to elevated temperatures, suggesting that GT incorporation had a beneficial effect on retaining the flexural performance of MAPC.

### 3.4. Variation in Mass Loss Rate

**Figure 9:**
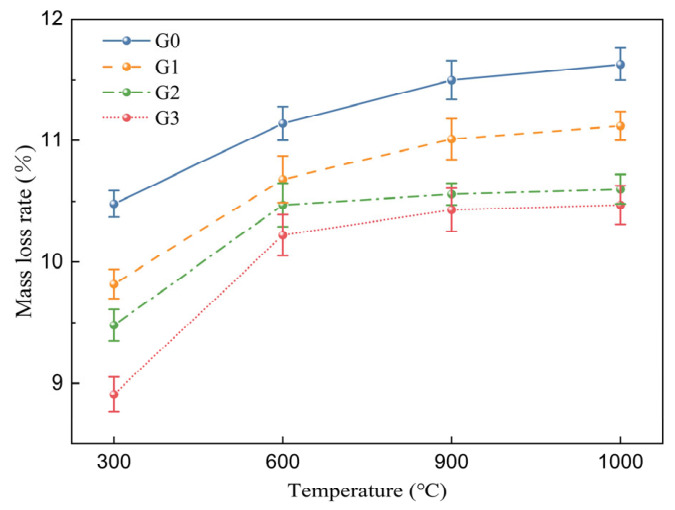
Mass loss rate of MAPC specimens.

The mass loss rate of MAPC at different temperatures is shown in Figure 9. Struvite, the main hydration product of MAPC, gradually loses water of crystallization and releases NH_3_ during heating. Its decomposition reaction can be expressed as follows [47]:

$${\mathrm{MgNH}}_{4}{\mathrm{PO}}_{4}\cdot 6H_{2}O(s)\;\to \;{\mathrm{MgHPO}}_{4}(s)\;+\;6H_{2}O↑\;+\;{\mathrm{NH}}_{3}↑$$

At 300 °C, all specimens exhibited marked mass loss rates, ranging from 8.91% to 10.48%, with G0 showing the highest value and G3 the lowest. The mass loss rate at this stage was mainly attributed to the dehydration and decomposition of struvite and the release of volatile components, such as NH_3_ and water of crystallization. This is consistent with the findings of Sugama et al. [1], who reported the rapid decomposition of struvite in the temperature range of 250–320 °C. The marked mass loss rate at this stage corresponded to the simultaneous decreases in compressive and flexural strengths, indicating that struvite decomposition and the release of volatile components may damage the original crystalline binding structure and promote the development of pores and microcracks.

When the temperature increased to 600 °C, the mass loss rate of each group increased by only 0.39–0.72% compared with that at 300 °C, indicating that the main decomposition process of struvite had been essentially completed near 300 °C. Thereafter, the increase in the mass loss rate became less pronounced, whereas the strength continued to decrease. This suggests that material deterioration in the medium- and high-temperature ranges was not governed solely by the mass loss rate, but was also associated with residual structural damage, pore expansion, and solid-phase structural adjustment [48]. At 900–1000 °C, the mass loss rate remained relatively stable, and the system evolution may have gradually shifted toward solid-phase transformation, reconstruction of magnesium phosphate phases, and the formation of locally sintered structures [49].

At the same temperature, the mass loss rate of MAPC generally decreased with increasing GT content. At 1000 °C, the mass loss rate of G3 was 9.84%, which was approximately 15% lower than that of G0. This was mainly because the equal-mass replacement of part of the total MAPC binder by GT reduced the relative content of decomposable struvite, thereby decreasing the mass loss caused by its dehydration and decomposition. Meanwhile, the silicoaluminous components in GT, such as SiO_2_ and Al_2_O_3_, exhibited a certain degree of stability at elevated temperatures and may have had a beneficial effect on maintaining the residual matrix after exposure to elevated temperatures [25].

### 3.5. XRD Phase Analysis

**Figure 10:**
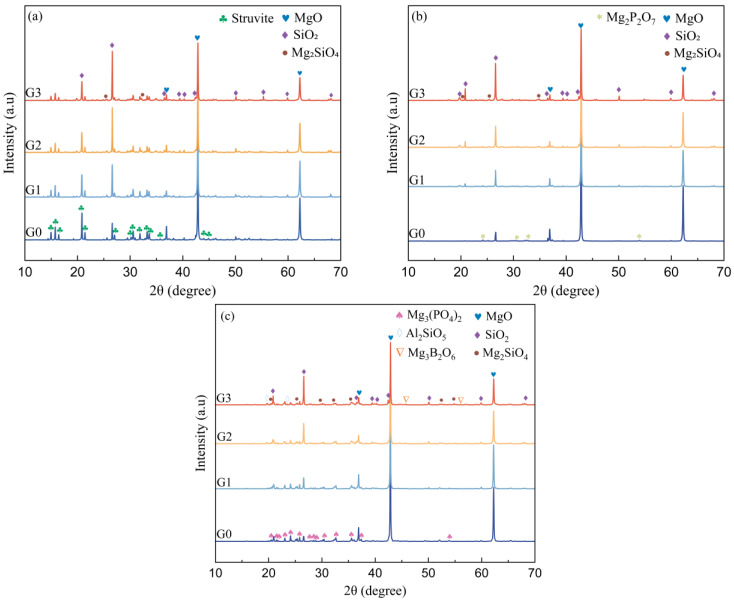
XRD patterns of MAPC specimens after treatment at different temperatures: (**a**) 25 °C, (**b**) 600 °C, and (**c**) 1000 °C.

Figure 10 shows the XRD patterns of MAPC after treatment at 25 °C, 600 °C, and 1000 °C. The strength of MAPC decreased markedly after treatment at 600 °C, whereas the mass loss rate was mainly concentrated below 300 °C and tended to level off after 600 °C. In addition, the specimen strength partially recovered at 1000 °C. Therefore, 25 °C, 600 °C, and 1000 °C were selected as representative temperatures for analyzing the phase evolution of MAPC by XRD.

#### 3.5.1. Qualitative Phase Identification

At 25 °C, as shown in Figure 10a, diffraction peaks assigned to struvite and MgO were detected in all specimens, indicating that struvite was the main hydration product of MAPC at room temperature. With increasing GT content, the peak intensity of struvite gradually decreased, suggesting that the equal-mass replacement of part of the total MAPC binder by GT reduced the formation of struvite. This result is consistent with the decrease in room-temperature strength. Meanwhile, diffraction peaks assigned to SiO_2_ and Mg_2_SiO_4_ were observed in the patterns, which were mainly associated with siliceous mineral components in the raw materials. The weak peaks observed in G0 may have originated from incomplete separation of quartz sand during powder sample preparation or from a small amount of siliceous impurities in the raw materials.

As shown in Figure 10b, the diffraction peaks of struvite almost disappeared at 600 °C, indicating that dehydration and decomposition had occurred and that the original crystalline structure had been disrupted. Previous studies have shown that, after decomposition at approximately 300 °C, struvite may form an amorphous magnesium phosphate structure based on Mg_2_P_2_O_7_, while Mg_2_P_2_O_7_ generally begins to crystallize appreciably only above approximately 690 °C [1,14]. Therefore, the weak Mg_2_P_2_O_7_ diffraction peaks at 600 °C indicate that the phosphate decomposition products at this stage mainly existed in low-crystallinity or amorphous forms, making it difficult to form a stable crystalline binding skeleton. This may be one of the important reasons for the continued strength reduction and appearance deterioration of the specimens at 600 °C. In addition, diffraction peaks assigned to MgO, SiO_2_, and Mg_2_SiO_4_ were still observed in the patterns at 600 °C. The relatively more distinct SiO_2_ and Mg_2_SiO_4_ peaks in the GT-containing specimens were mainly related to the siliceous mineral components introduced by GT.

When the temperature increased to 1000 °C, as shown in Figure 10c, diffraction peaks assigned to Mg_3_(PO_4_)_2_ appeared in the system. Wang et al. [50] reported that MAPC can gradually transform from struvite to Mg_3_(PO_4_)_2_ at elevated temperatures and undergo local sintering at 1000 °C and above, thereby improving the mechanical strength and thermal stability of the residual matrix. Therefore, the formation of Mg_3_(PO_4_)_2_ and the development of locally sintered structures may be important reasons for the recovery in specimen strength. In addition, diffraction peaks assigned to Mg_3_B_2_O_6_ were detected, indicating that MgO and borax may have reacted at elevated temperatures. In the GT-containing specimens, the diffraction peaks of Mg_2_SiO_4_ became more pronounced, and diffraction peaks related to Al_2_SiO_5_ appeared, suggesting that the siliceous and silicoaluminous components introduced by GT may have participated in the high-temperature phase evolution. The formation of thermally stable phases, such as Mg_2_SiO_4_ and Al_2_SiO_5_, may contribute to improving the thermal stability of the residual matrix, which is consistent with the higher strength retention of the GT-containing specimens at 1000 °C.

#### 3.5.2. Semi-Quantitative Phase Analysis

**Figure 11:**
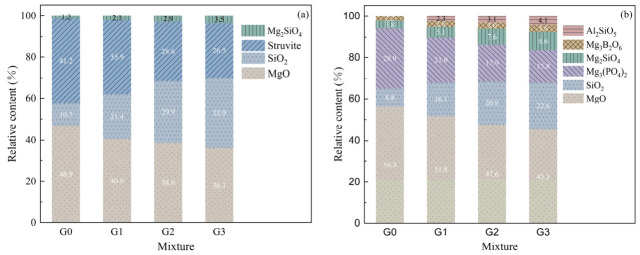
Relative contents of the main phases in MAPC specimens: (**a**) 25 °C and (**b**) 1000 °C.

Whole-pattern fitting (WPF) and Rietveld refinement were performed on the XRD patterns of MAPC at 25 °C and 1000 °C using Jade 9.0 to obtain the relative contents of the main phases in each group. The results are shown in Figure 11. Because low-crystallinity or amorphous phases may exist in the system after high-temperature treatment and some diffraction peaks overlap, the phase contents shown in Figure 11 should be regarded as semi-quantitative results and used mainly to compare the relative variation trends among different groups.

As shown in Figure 11a, the relative content of struvite in G0 was 41.2% and gradually decreased with increasing GT content. This trend is consistent with the decrease in room-temperature strength, indicating that GT incorporation reduced the formation of the struvite binding phase. In addition, the relative contents of Mg_2_SiO_4_ and SiO_2_ in the GT-containing specimens increased with increasing GT content, reflecting the increased proportion of siliceous components in the system. It should be noted that quartz sand mainly acts as a filler in MPC systems and has a limited direct contribution to chemical densification [37]. In contrast, the presence of unreacted MgO can provide skeleton support to some extent [1,33].

After treatment at 1000 °C, as shown in Figure 11b, the phase composition of the system changed markedly. Compared with that at 25 °C, the relative content of SiO_2_ decreased, whereas that of Mg_2_SiO_4_ increased, indicating that part of the siliceous components may have participated in high-temperature solid-state reactions. Meanwhile, the relative contents of Mg_3_B_2_O_6_ and Al_2_SiO_5_ increased with increasing GT content, suggesting that GT modified the high-temperature phase composition of the system to some extent, which may be beneficial for improving the mechanical properties of MAPC after exposure to elevated temperatures.

### 3.6. SEM Microstructural Analysis

**Figure 12:**
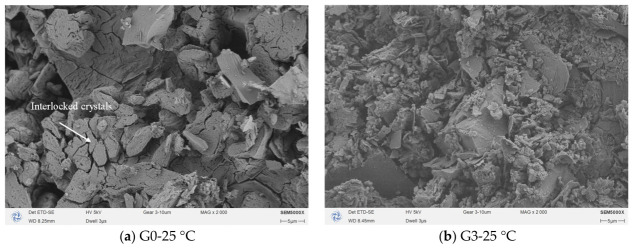

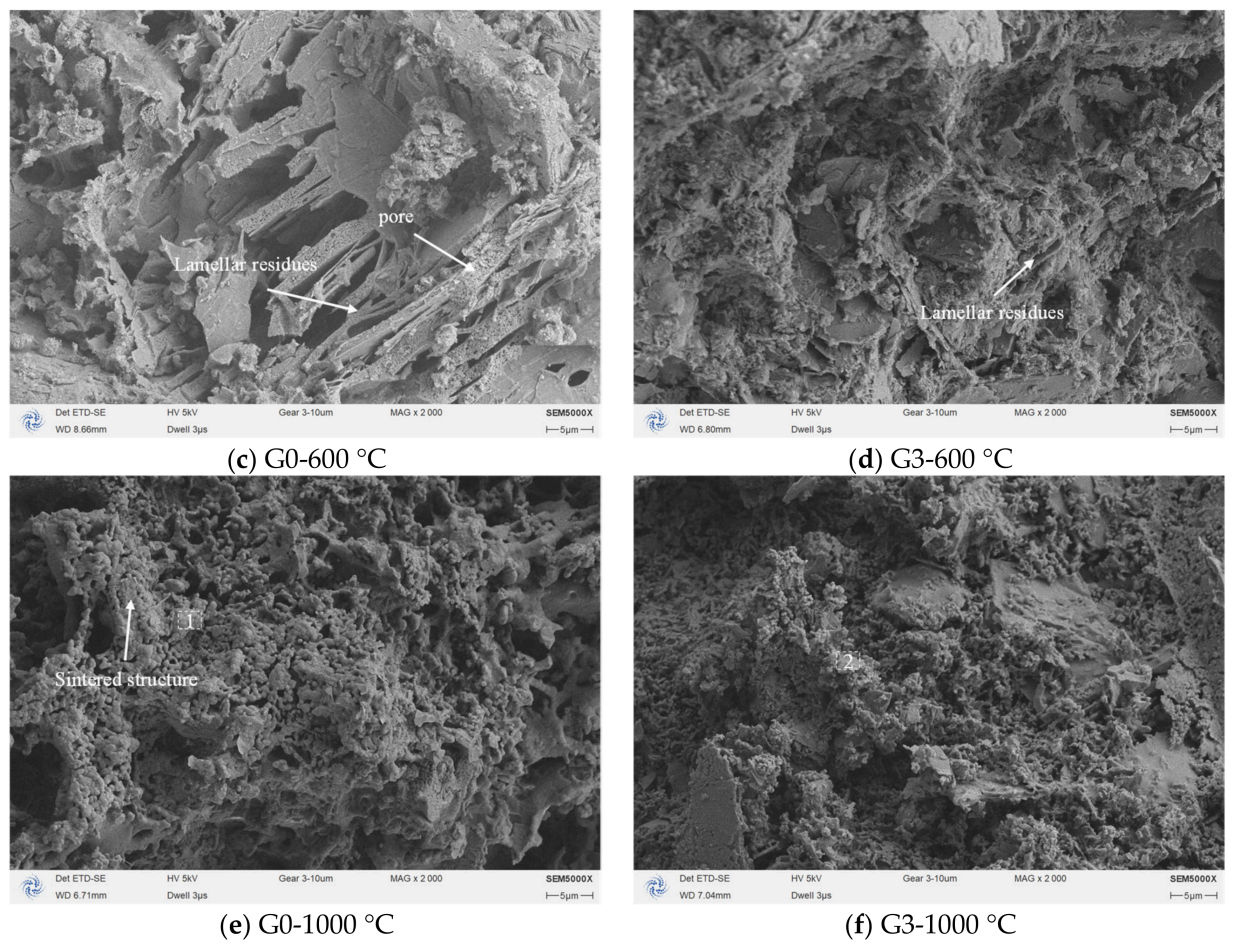
SEM images of MAPC specimens after treatment at different temperatures: (**a**) G0 at 25 °C; (**b**) G3 at 25 °C; (**c**) G0 at 600 °C; (**d**) G3 at 600 °C; (**e**) G0 at 1000 °C; (**f**) G3 at 1000 °C.

**Figure 13:**
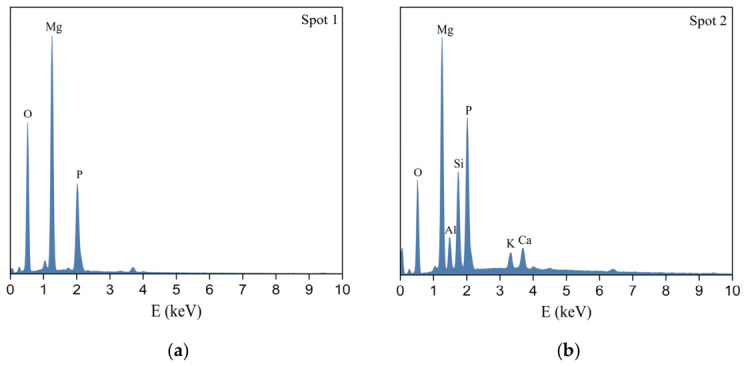
EDS point analysis results of MAPC specimens after treatment at 1000 °C: (**a**) Spot 1; (**b**) Spot 2.

To compare the microstructural differences between the specimen without GT and the specimen with the highest GT content, G0 and G3 were selected as representative groups for SEM analysis. Figure 12 shows the typical fracture surface morphologies of G0 and G3 after treatment at 25 °C, 600 °C, and 1000 °C, and Figure 13 presents the EDS point analysis results of representative regions at 1000 °C.

As shown in Figure 12a,b, numerous flaky or blocky hydration products were observed in G0 at 25 °C. These crystals overlapped and interlocked with each other, forming a relatively continuous binding skeleton. The main hydration product of MAPC at room temperature is struvite, whose flaky or plate-like morphology is consistent with that reported in previous studies and constitutes the main binding phase of MAPC [1,48]. In G3, more granular substances and local agglomerates were observed, and the continuity of the crystalline binding skeleton was reduced. This observation is consistent with the lower room-temperature strength of G3 compared with that of G0.

At 600 °C, as shown in Figure 12c,d, the original crystalline binding structure in G0 was markedly disrupted, and pores, cracks, and lamellar residual structures were observed within the matrix. This is consistent with the disappearance of the struvite diffraction peaks and the weak Mg_2_P_2_O_7_ diffraction peaks in the XRD results, indicating that the dehydration and decomposition of struvite and the release of volatile components caused microstructural deterioration. This observation also corresponds to the continued decrease in strength at this stage [42,47,51]. The fracture surface morphology of G3 differed from that of G0, with granular residues locally attached to the matrix surface.

After treatment at 1000 °C, as shown in Figure 12e,f, relatively dense, locally sintered structures were observed in G0, indicating that the residual phases underwent a certain degree of reconstruction and particle bonding at elevated temperatures. This provides microstructural evidence for the strength recovery of the specimens at 1000 °C [48,52,53]. The EDS point analysis result of Spot 1 in Figure 13 shows that this region was mainly composed of Mg, P, and O. Combined with the phase analysis results, this region can be inferred to be associated with magnesium phosphate residual phases, such as Mg_3_(PO_4_)_2_, formed at high temperature. The EDS point analysis result of Spot 2 shows that, in addition to Mg, P, and O, Si, Al, and small amounts of K and Ca were also detected, indicating the presence of Si- and Al-rich regions in the GT-containing specimen. At 1000 °C, Mg_3_(PO_4_)_2_, Mg_2_SiO_4_, and aluminosilicate phases coexisted in G3, indicating that GT modified the high-temperature residual phase composition of MAPC. The formation of these thermally stable phases and locally sintered structures may contribute to improving the residual matrix structure after exposure to elevated temperatures and provides a microstructural explanation for the enhanced strength retention of the GT-containing specimens.

## 4. Conclusions

In this study, the effects of GT incorporation on the workability, visual appearance, mechanical properties, mass loss rate, phase evolution, and microstructure of MAPC mortar were investigated. The main conclusions are as follows:

1. GT incorporation shortened the setting time of MAPC and reduced its fluidity. These changes may be related to MgO dissolution, variations in the retarding effect, the irregular morphology of GT particles, and increased interparticle friction.
2. The surface color of MAPC specimens was mainly governed by temperature and changed noticeably with increasing temperature. At the same temperature, the GT-containing specimens appeared to exhibit less severe surface deterioration, which was beneficial for maintaining the visual integrity of the specimens after exposure to elevated temperatures.
3. At room temperature, the mechanical strength of MAPC decreased with increasing GT content, mainly owing to the reduced formation of the struvite binding phase and the decreased continuity of the crystalline binding skeleton. After exposure to elevated temperatures, MAPC exhibited marked strength degradation in the range of 300–600 °C. At 900 °C and above, the GT-containing specimens showed higher strength retention. At 1000 °C, the compressive strength of G3 was 15.37 MPa, which was approximately 44.0% higher than that of G0, and its flexural strength retention reached 47.42%.
4. The thermal decomposition of struvite and the release of volatile components, such as NH_3_ and water of crystallization, were important causes of the increased mass loss rate and strength deterioration of MAPC. At 1000 °C, the formation of thermally stable phases, including Mg_3_(PO_4_)_2_, Mg_2_SiO_4_, and aluminosilicates, may have contributed to the recovery in strength.
5. Within the experimental scope of this study, GT incorporation improved the residual mechanical properties of MAPC after exposure to elevated temperatures, and the specimen containing 30% GT showed comparatively superior performance. A GT content of 20–30% can be considered as a reference range for the further optimization of MAPC-based heat-resistant repair materials.

## Figures and Tables

**Table 1:** Chemical compositions of the main raw materials (%).

Raw Material	SiO_2_	Al_2_O_3_	Fe_2_O_3_	K_2_O	MgO	CaO	Na_2_O	Others
GT	68.51	17.20	5.07	4.90	1.57	1.41	0.96	0.38
MgO	0.65	0.21	0.16	-	96.68	2.06	-	0.24

**Table 2:** Mix proportions of MAPC mortar specimens.

Mixture	Binder Composition (%)			B/M	W/C	S/C
	MgO	NH_4_H_2_PO_4_	GT			
G0	80	20	0	0.05	0.18	1
G1	72	18	10			
G2	64	16	20			
G3	56	14	30			

## Data Availability

The original contributions presented in this study are included in the article. Further inquiries can be directed to the corresponding author.

## References

[1] Sugama T., Kukacka L. (1983). Magnesium monophosphate cements derived from diammonium phosphate solutions. Cem. Concr. Res..

[2] Walling S.A., Provis J.L. (2016). Magnesia-based cements: A journey of 150 years, and cements for the future?. Chem. Rev..

[3] Dabarera A., Fernández R., Provis J.L. (2024). A systematic review of engineering properties of magnesium potassium phosphate cement as a repair material. Front. Mater..

[4] Xu B., Lothenbach B., Winnefeld F. (2020). Influence of wollastonite on hydration and properties of magnesium potassium phosphate cements. Cem. Concr. Res..

[5] Li Y., Sun J., Chen B. (2014). Experimental study of magnesia and M/P ratio influencing properties of magnesium phosphate cement. Constr. Build. Mater..

[6] Zhang X., Li G., Niu M., Song Z. (2018). Effect of calcium aluminate cement on water resistance and high-temperature resistance of magnesium-potassium phosphate cement. Constr. Build. Mater..

[7] Tan Y., Gardner L.J., Walkley B., Hussein O.H., Ding H., Sun S., Yu H., Hyatt N.C. (2023). Optimization of magnesium potassium phosphate cements using ultrafine fly ash and fly ash. ACS Sustain. Chem. Eng..

[8] Chhaiba S., Martinez-Sanchez S., Husillos-Rodriguez N., Palomo Á., Kinoshita H., Garcia-Lodeiro I. (2024). Durability of magnesium potassium phosphate cements (MKPCs) under chemical attack. Materials.

[9] Soudée E., Péra J. (2000). Mechanism of setting reaction in magnesia-phosphate cements. Cem. Concr. Res..

[10] Yang Y., Liu Y., Yan Z., Chen Z. (2022). High-temperature resistance of modified potassium magnesium phosphate cement. Materials.

[11] Yu J., Qian J., Chen H., Ji Y., Kuang D., Jia X., Guan B. (2023). Behavior of magnesium phosphate cement with addition of sulphoaluminate cement at elevated temperatures. Constr. Build. Mater..

[12] Dai X., Zhang P., Gao Z., Qian J. (2025). Improving volume stability of magnesium phosphate cement at high temperatures by adding alumina-bearing admixtures. Constr. Build. Mater..

[13] Li Y., Shi T., Chen B., Li Y. (2015). Performance of magnesium phosphate cement at elevated temperatures. Constr. Build. Mater..

[14] Neiman R., Sarma A.C. (1980). Setting and thermal reactions of phosphate investments. J. Dent. Res..

[15] Runqing L., Wei W., Dingwen Q., Yuanquan Y. (2023). Static and dynamic mechanical properties of magnesium phosphate cement modified by metakaolin after high-temperature treatment. Constr. Build. Mater..

[16] Fang Y., Cui P., Ding Z., Zhu J.-X. (2018). Properties of a magnesium phosphate cement-based fire-retardant coating containing glass fiber or glass fiber powder. Constr. Build. Mater..

[17] Çelik Ö., Elbeyli I.Y., Piskin S. (2006). Utilization of gold tailings as an additive in Portland cement. Waste Manag. Res..

[18] Araujo F.S., Taborda-Llano I., Nunes E.B., Santos R.M. (2022). Recycling and reuse of mine tailings: A review of advancements and their implications. Geosciences.

[19] Zhao Z., Ji C., Wang J., Zhu L., Wang D., Tošić N. (2025). Investigation of gold mine tailings as supplementary cementitious material: Performance and carbon footprint. J. Clean. Prod..

[20] Tapfuma A., Mutimutema P., Bazhko O., Nkabinde S., Ledwaba M., Kabangu J.M., Chiwaye N., Muller E., Ndlovu G. (2026). Gold tailing metal extraction and sustainable end-use: A closed-loop review. Miner. Eng..

[21] Zhang Y., Wan Z., Wang L., Guo B., Ma B., Chen L., Tsang D.C. (2022). Designing magnesium phosphate cement for stabilization/solidification of Zn-rich electroplating sludge. Environ. Sci. Technol..

[22] Padin J.M.B., Balayo K.L.P., Rapanot E.M.S., Logrosa G.T. (2026). Material reusability in mine waste: A review on utilization of gold ore tailings in concrete production. Sustain. Chem. One World.

[23] Xu C., Han J., Yang Y. (2024). A review on magnesium potassium phosphate cement: Characterization methods. J. Build. Eng..

[24] Meng X., Jiang Y., Chen B., Wang L. (2023). Research progress on the setting time and solidification mechanism of magnesium phosphate cement: A review. Constr. Build. Mater..

[25] Zhang Z., Ruan W., He X., Cai M., Liu J., She Y., Yan M., Li K., Liao J. (2024). Comparative study on the effect of bauxite tailings on properties of magnesium-phosphate-cement-based materials before and after calcination. Constr. Build. Mater..

[26] Zhu Y., Wang Z., Li Z., Yu H. (2022). Experimental research on the utilization of gold mine tailings in magnesium potassium phosphate cement. J. Build. Eng..

[27] Qin L., Xie Q., Yang J., Bao J., Song Q., Wang S., Yu Q., Niu D., Zhang P. (2024). Effect of coal fly ash and CO2 curing on performance of magnesium potassium phosphate cement. J. CO_2_ Util..

[28] Gelli R., Tonelli M., Martini F., Calucci L., Borsacchi S., Ridi F. (2022). Effect of borax on the hydration and setting of magnesium phosphate cements. Constr. Build. Mater..

[29] (2011). Test Methods for Water Requirement of Normal Consistency, Setting Time and Soundness of Portland Cement.

[30] (2019). Magnesium Phosphate Repairing Mortar.

[31] (1999). Method of Testing Cements—Determination of Strength.

[32] Xu B., Winnefeld F., Kaufmann J., Lothenbach B. (2019). Influence of magnesium-to-phosphate ratio and water-to-cement ratio on hydration and properties of magnesium potassium phosphate cements. Cem. Concr. Res..

[33] Li Y., Chen B. (2013). Factors that affect the properties of magnesium phosphate cement. Constr. Build. Mater..

[34] Xu B., Lothenbach B., Leemann A., Winnefeld F. (2018). Reaction mechanism of magnesium potassium phosphate cement with high magnesium-to-phosphate ratio. Cem. Concr. Res..

[35] Le Rouzic M., Chaussadent T., Platret G., Stefan L. (2017). Mechanisms of k-struvite formation in magnesium phosphate cements. Cem. Concr. Res..

[36] Zhang T., Chen H., Li X., Zhu Z. (2017). Hydration behavior of magnesium potassium phosphate cement and stability analysis of its hydration products through thermodynamic modeling. Cem. Concr. Res..

[37] Yue Y., Ren J., Yang K., Wang D., Qian J., Bai Y. (2022). Investigation and optimisation of the rheological properties of magnesium potassium phosphate cement with response surface methodology. Materials.

[38] Zhang H., Zhang Q., Tang S., Pei Y., Skoczylas F. (2024). Effects of fly ash and silica fume on the rheological properties of magnesium phosphate cement-emulsified asphalt (MPC-EA) composite repair materials. Constr. Build. Mater..

[39] Gao X., Zhang A., Li S., Sun B., Zhang L. (2016). The resistance to high temperature of magnesia phosphate cement paste containing wollastonite. Mater. Struct..

[40] Gardner L., Lejeune V., Corkhill C., Bernal S., Provis J., Stennett M., Hyatt N. (2015). Evolution of phase assemblage of blended magnesium potassium phosphate cement binders at 200 and 1000 °C. Adv. Appl. Ceram..

[41] Qian W., Sun W., Li R., Wang J., Zhan S. (2024). Study on the high temperature performance of magnesium and potassium phosphate cement mixed with sucrose and its mechanism of action. Constr. Build. Mater..

[42] Frost R., Weier M., Erickson K.L. (2004). Thermal decomposition of struvite. J. Therm. Anal. Calorim..

[43] Sierra-Beltran M.G., Jonkers H., Schlangen E. (2014). Characterization of sustainable bio-based mortar for concrete repair. Constr. Build. Mater..

[44] Sarkar A. (1991). Hydration/dehydration characteristics of struvite and dittmarite pertaining to magnesium ammonium phosphate cement systems. J. Mater. Sci..

[45] Gardner L.J., Walling S.A., Corkhill C.L., Bernal S.A., Lejeune V., Stennett M.C., Provis J.L., Hyatt N.C. (2021). Temperature transformation of blended magnesium potassium phosphate cement binders. Cem. Concr. Res..

[46] Liu Y., Chen Z., Ni H., Liu K., He J. (2024). High-temperature properties of fly ash and silica fume composite magnesium potassium phosphate cement. Constr. Build. Mater..

[47] Lavanya A. (2026). Investigation of thermal degradation pathway and kinetics of struvite recovered from dairy wastewater via thermogravimetric studies. J. Therm. Anal. Calorim..

[48] Lahalle H., Coumes C.C.D., Mesbah A., Lambertin D., Cannes C., Delpech S., Gauffinet S. (2016). Investigation of magnesium phosphate cement hydration in diluted suspension and its retardation by boric acid. Cem. Concr. Res..

[49] Lahalle H., Coumes C.C.D., Mercier C., Lambertin D., Cannes C., Delpech S., Gauffinet S. (2018). Influence of the w/c ratio on the hydration process of a magnesium phosphate cement and on its retardation by boric acid. Cem. Concr. Res..

[50] Wang N., Bi W., Guo F., Guan Y., Li J. (2024). Effect of silica fume on the properties of magnesium ammonium phosphate cement at room temperature and high temperatures. J. Mater. Civ. Eng..

[51] Hefney S., Awotoye D.T., Fairley N., Laskin A., Baltrusaitis J. (2026). In Situ ATR-FTIR Nonisothermal Kinetic Analysis of Struvite–Dittmarite Thermal Transformation. ACS Earth Space Chem..

[52] Preobrazhenskiy I.I., Deyneko D.V., Murashko A.M., Klimashina E.S., Filippov Y.Y., Evdokimov P.V., Putlayev V.I. (2025). Ceramic materials based on magnesium orthophosphate for biomedical applications. Mendeleev Commun..

[53] Zhang S., Li L., Lv X. (2017). Synthesis and characterization of a novel Mg_3_(PO_4_)_2_ ceramic with low dielectric constant. J. Mater. Sci. Mater. Electron..

